# Applying SF-6D to measure health state utilities among the middle and old aged patients with hypertension in China

**DOI:** 10.1186/s12955-020-01598-4

**Published:** 2020-12-11

**Authors:** Xiaohan Liu, Guannan Bai, Hui Li, Shunping Li

**Affiliations:** 1 Centre for Health Management and Policy Research, School of Health Care Management, Cheeloo College of Medicine, Shandong University, Wenhua Xi Rd 44, Jinan, 250012 Shandong Province People’s Republic of China; 2 NHC Key Lab of Health Economics and Policy Research, Shandong University, Jinan, 250012 People’s Republic of China; 3 Department of Public Health, Erasmus MC-University Medical Center, Wytemaweg 80, Rotterdam, 3015CN The Netherlands; 4 Centre for Quality of Life and Public Policy Research, Shandong University, Jinan, 250012 People’s Republic of China

**Keywords:** Health utility, Hypertension, Preference-based measurement, Quality of life, SF-6D

## Abstract

**Purpose:**

Hypertension is a growing public health problem in China; however, little is known about health-related quality of life (HRQoL) especially health state utility (HSU) of patients with hypertension in rural China. This study aimed to examine the HSU as measured by SF-6D and to investigate its associated factors among middle and old aged patients with hypertension in rural China.

**Methods:**

Data were collected from twelve villages in Shandong Province in 2016. SF-36 was administrated to measure HRQoL of middle and old aged patients with hypertension and was got to the SF-6D values using Hong Kong’s tariff. Descriptive analyses, such as demographic characteristics, socio-economic status, and utility, were stratified by hypertension classification. Multiple linear regression models were applied to assess the associated factors of HSU.

**Results:**

A total of 933 (response rate:86.4%) middle and old aged patients (69.1 ± 8.2 years) with hypertension participated in the study. 39.4% of participants were male; 44.2% had stage I hypertension; 26.4% had stage II and above. The mean score of SF-6D utility score was 0.743 (SD: 0.14, range: 0.32–1.00, median: 0.756, Interquartile range:0.634–0.859). Being female (β = −0.046), having two or more comorbidities (2 vs. 1 β = −0.066; > 3 vs. 1 β = −0.098) and the health expenditure higher than 2000 RMB (2000–3999 vs.< 2000 β = −0.042; 4000–5999 vs. < 2000 β = −0.046; > 6000 vs. < 2000 β = −0.071) were significantly associated with lower SF-6D overall score; while being farmer (β = 0.032), having high household income (10,000–14,999 vs. < 5000 β = 0.045; > 15,000 vs. < 5000 β = 0.064) and having stage I and above hypertension (stage I vs. Normotensive β = 0.047; stage II vs. Normotensive β = 0.079; stage III vs. Normotensive β = 0.095) were significantly associated with higher SF-6D overall score.

**Conclusion:**

SF-6D was capable to measure quality of life middle and old aged patients with hypertension in China. And multiple factors were demonstrated to be significantly associated with quality of life.

## Introduction

Hypertension is the strongest risk factor of cardiovascular and cerebrovascular diseases as well as the related disabilities and mortality in worldwide [1]. About 80% of all cardiovascular mortality occurs in low-income and middle-income countries including China, where the greatest burden of hypertension has been observed [2]. A recent nation-wide survey in China showed that nearly half of Chinese adults aged 35–75 years had hypertension, and among them, a third had stage II and above hypertension [3]; more than two-thirds of the survey population were not treated, and fewer than one in twelve were in control of their blood pressure. Health-related quality of life (HRQOL) of individuals with hypertension was lower than normotensive individuals [4–7]. In China, rural population account for over 40% of the whole population, while in Shandong province, 60% of the population are in the rural areas [8]. The prevalence of hypertension continues to rise in rural areas and has surpassed that in urban area [3]. However, rural people were less likely to be aware of, treated for, and controlled for hypertension [3, 9]. Therefore, as a chronic non-communicable disease, hypertension has unsatisfactory morbidity and control in China rural, influencing seriously hypertension patients’ quality of life, which warranted further research.

HRQoL is a multidimensional concept encompassing a patient’s emotional, physical, and social functioning [10]. Various instruments of HRQOL have been developed for the general population and populations with health conditions including hypertension [11, 12]. Based on the literature review, most of the previous studies measured HRQoL of hypertension using the generic instruments, such as MOS (Medical Outcome Study) and 36-Item Short-Form Health Survey (SF-36), while few studies used the preference-based measurements among patients with hypertension [13–15].

Health state utility (HSU) is essential to cost-utility analysis (CUA) in health administration and health economics [16]. The HSU scores can be elicited using multi-attributes utility (MAU) instruments, such as the EuroQol-5 Dimensions (EQ-5D) instrument, the Short-Form Six Dimensions (SF-6D) derived from the SF-36, and the Health Utilities Index (HUI). With regard to the preference-based instruments of HRQOL in patients with hypertension, there have been some studies applying EQ-5D [5, 7, 17–20] and HUI [21, 22]. To our best knowledge, only one study conducted in Vietnam used SF-6D to evaluate the utility value of patients with hypertension [23]. There has been no similar data in China.

In addition, identifying the associated factors especially the modifiable factors are important for further development of evidence-based health promotion and intervention programs among rural population in China. Evidence on this issue was scarce. Based on the existing literature, age, gender, marital status, income, education level, awareness of hypertension, and comorbidities were associated with quality of life of patients with hypertension [5]. However, little is known about the associated factors of quality of life among patients in rural areas in China.

Therefore, this study aimed to evaluate the performance of SF-6D as a measurement of health status utility and to assess the associated factors of quality of life among middle and old aged patients with hypertension in rural areas, China. This study would benefit the future studies in China or in other countries by providing the reference data of SF-6D of patients with hypertension for comparison with other populations.

## Methods

### Setting and sample

This study was conducted in Shandong Province, which is located in middle east of China, with a population of about 100 million [24]. In 2016, the gross regional product of Shandong Province amounted to RMB 6802 billion (US$1024 billion), ranking as the third-largest economy in China [24]. It illustrates the Chinese economy and society in miniature to some extent. Like China, Shandong also presents a gradient regarding the level of economic development across the east, middle and west areas, that is, the economy in the east is better than the other two areas. We followed the randomized sampling principles to optimize the representativeness of the study population by selecting villages from different geographic areas. In addition, the economy, society and culture do not diverse much in rural areas in Shandong Province. Hence, the study population in our study has a relatively good representativeness.

A community-based survey was conducted in July, 2016. We applied the method of stratified random sampling to select the study population. Three tiers were used. To ensure the representation of our sample, firstly, we selected three cities in the east, middle and west of Shandong Province. Secondly, from these three cities, we selected three counties based on their economic level, i.e. high, middle and low development of economy. Thirdly, two villages were selected randomly from each county. Two townships were then selected randomly from each county, and two villages were further randomly selected from each township. Finally, twelve villages were enrolled.

In 2009, the New Health System Reform was launched in China, and all diagnosed patients with hypertension should be registered and managed by village doctors according to the Basic Public Health Services Regulation 2009 [25]. In this study, we recruited all patients with hypertension aged 50 years and above in these twelve villages based on the registration at the village doctors. Patients were excluded if (1) they were unwilling to give informed consent; (2) they had the disease that severely influenced the cognitive function to comprehend the questionnaire; or (3) they did not register at the village doctors. In total, one thousand and eighty patients were eligible for the survey. One hundred and twenty-six patients were excluded because they were unavailable for the interview. Further, twenty-one patients were excluded due to incomplete answers to SF-36. The final study sample consists of 933 participants (response rate: 86.4%).

### Instruments

Twenty-two interviewers were recruited among the medical ungraduated and graduate students at Shandong University. The interviewers were trained several times by the project management team members with regard to the study protocol, targeted population, questionnaire, interview skills, recording data, and the proper method to measure hypertension. They interviewed the participants in a face-to-face way. To control the quality of the questionnaire, interviewers carefully checked the questionnaires at the end of the day, and if there were any questions, they asked investigators to clarify.

#### HRQOL measurement

HRQOL was measured by the Chinese version of SF-36 [28], from which the SF-6D was derived [29]. For more information about SF-36, see the links: https://www.qualitymetric.com/products/license/; and https://www.rand.org/health/surveys_tools/mos/36-item-shortform.html. SF-6D includes six dimensions, i.e., physical functioning, role limitations, social functioning, pain, mental health, and vitality. There are four to six answer options of each dimension, resulting in 18,000 possible health states [29]. The SF-6D has been used to calculate health utility scores in terms of non-communicable diseases [30, 31]. In the present study, we used the Chinese-specific tariff developed among Chinese adults in Hong Kong to get SF-6D values from SF-36 [32]. The range of the overall score is 0.32–1.00 [29]. The higher score indicates better health status.

#### Hypertension measurement

 Blood Pressure (BP) was measured using an electronic sphygmomanometer (OMRON, HEM-7211). According to the Chinese guideline of the management of hypertension [27], the blood pressure was divided into four categories: normotensive hypertension, hypertension stage I, II, and III. Normative hypertension was defined as systolic blood pressure < 140 mmHg or diastolic blood pressure < 90 mmHg; Hypertension stage I: 140 mmHg ≤ systolic blood pressure < 160 mmHg or 90 mmHg ≤ diastolic blood pressure < 100 mmHg; Hypertension stage II: 160 mmHg ≤ Systolic blood pressure < 180 mmHg or 100 mmHg ≤ diastolic blood pressure < 110 mmHg; Hypertension stage III: Systolic blood pressure ≥ 180 mmHg or diastolic blood pressure ≥ 110 mmHg [27].

### Associated factors

A standardized questionnaire was designed to collect data, including socio-demographic information on the individuals and households, health-related behaviors, health conditions (hypertension and its complications, family history, and the economic burden of hypertension), quality of life, and utilization of health services. Based on the previous studies [19, 20, 33], we measured the potential associated factors of quality of life of hypertension patients, including socio-demographic factors, lifestyle-related factors, and the medical treatments of hypertension These factors were demonstrated to be significantly associated with the good/poor quality of life of hypertension patients in the previous studies.

### Data collection

#### Questionnaire survey

During the questionnaire survey, the trained-interviewers informed the participants about the basic principles of the survey and gave some instruction about how to fill in the questionnaire. They provided help when the participants had any questions. When the questionnaire was finished, the interviewer immediately checked the missing answers, and asked the participate to fill in again**.**

#### Hypertension measurement

Two blood pressure (BP) measurements were obtained from each participant by trained and certified observers according to a common protocol. Participants in the sitting position after 5 min of rest. Participants were instructed to refrain from alcohol, cigarette smoking, coffee/tea, and exercise for at least 30 min before their BP measurement. The average of two or three readings of systolic BP and Diastolic BP was used to describe each participant’s BP. It’s worth noting that some participants showed normal blood pressure when they participate in the survey because they had taken measures to control their hypertension, such as taking regular medication.

### Statistical analysis

We described the frequencies of options of each item of SF-6D answered by participants. Kolmogorov–Smirnov test (K–S test) was applied to evaluate the distribution of SF-6D scores.

Differences in the overall score of SF-6D across the subgroups regarding the variables of attributes of the study population were assessed by one-way analysis of variance (ANOVA) when the variables were normally distributed, otherwise the Mann–Whitney *U* rank-sum test was used. Multivariate linear regression was applied to assess the associated factors of SF-6D overall score. The statistical significance level was indicated as p < 0.01. All statistical analyses were performed using SPSS statistics (SPSS version 22.0 Inc., Chicago, IL, USA).

### Ethics approval

This study was conducted according to the guideline proposed in the World Medical Association of Helsinki [26], and the ethical approval was obtained from the Ethics Review Board of the School of Public Health, Shandong University (Reference No. 20140301). Informed consent was obtained from all participants after a detailed explanation of the study by the interviewers.

## Results

### Participants’ characteristics

Table 1 presented the characteristics of the participants. The mean age of the study population was 69.1 (SD = 8.2) years old. About 40% of the participants were male and about half of the population were illiterate (48.3%). More than 80% participants were farmers and more than half had household income of less than 10,000 RMB a year. Nearly 70% of the participants had hypertension for more than 5 years. The majority (more than 80%) of non-drug therapy patients followed alimentary control, work-rest schedule and rest, and emotion control. In terms of drug compliance, the condition that patients' discontinuation medication by themselves was more serious. Almost one-third of participants were classified as normotensive and around 45% were in stage I, 26.4% were above the stage II.

**Table 1:** Participants’ characteristics (n = 933)

Variables	N (%)
*Gender *	
Male	368 (39.4)
Female	565 (60.6)
Age*	
50–59	86 (9.2)
60–69	398 (42.7)
70–79	349 (37.4)
> 80	76 (8.1)
*Education *	
Illiteracy	451 (48.3)
Primary	278 (29.8)
Junior	160 (17.1)
Senior or above	44 (4.7)
*Marital status *	
Married	781 (83.7)
Others	152 (16.3)
*Way of residence *	
Living alone	115 (12.3)
Living with a spouse	587 (62.9)
Others	231 (24.8)
*Occupation *	
Farmers	763 (81.8)
Others	170 (18.2)
Household income (RMB)*	
< 5000	342 (36.7)
5000–9999	176 (18.9)
10,000–14,999	135 (14.5)
> 15000	280 (30.0)
Health expenditure (RMB)*	
< 2000	390 (41.8)
2000–3999	95 (10.2)
4000–5999	117 (12.5)
> 6000	250 (26.8)
*Comorbid diseases *	
1	552 (59.2)
2	301 (32.3)
> 3	80 (8.6)
*Health insurance *	
No	26 (2.8)
Yes	907 (97.2)
*Hypertension stages *	
normotensive	274 (29.4)
I	412 (44.2)
II	172 (18.4)
III	75 (8.0)
*Duration of hypertension (Years) *	
< 5	318 (34.1)
5–9	271 (29.0)
10–14	153 (16.4)
> 15	191 (20.5)
*Non-pharmaceutical therapies *	
*Exercise *	
No	467 (50.5)
Yes	466 (49.9)
*Alimentary control *	
No	154 (16.5)
Yes	779 (83.5)
*Work-rest schedule *	
No	154 (16.5)
Yes	779 (83.5)
*Emotion control *	
No	136 (14.6)
Yes	797 (85.4)
*Pharmaceutical therapies *	
*Take medicine in one year *	
No	112 (12.0)
Yes	821 (88.0)
*Take medicine in two weeks *	
No	209 (22.4)
Yes	724 (77.6)
*Pharmaceutical Compliance *	
Forget taking medicine *	
No	540 (63.6)
Yes	309 (36.4)
Drug discontinuation by patients themselves*	
No	699 (82.3)
Yes	150 (17.7)
Drug discontinuation for improvement*	
No	550 (64.8)
Yes	299 (35.2)
Drug discontinuation for deterioration*	
No	698 (82.2)
Yes	151 (17.8)

*Note*: OECD Health Statistics 2018: (https://www.oecd.org/els/health-systems/health-data.htm)

Monetary conversion rate: US$1 = RMB 6.2(2015) US$1 = RMB 6.6(2016); US$1 = RMB 6.8(2017);

Others in Marital status: unmarried, divorced, widowed, etc.

Others in Way of residence: live with friends, other relatives, aunts and uncles, children, etc.

Others in Occupation: Work in a public institution such as government, hospital, school, etc. work in corporations, work in individual business etc.

^*^Missing data

**Fig. 1:**
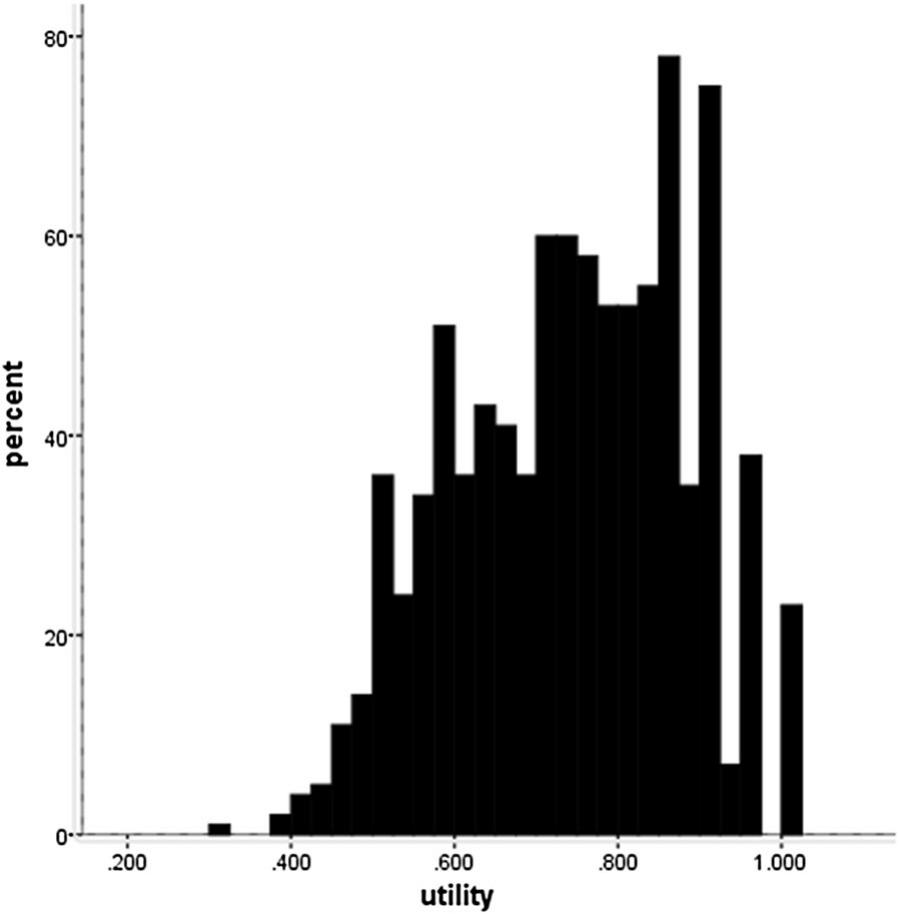
Distribution of SF-6D utilities

Table 2 showed the frequencies of each level of six dimensions of SF-6D: 47.3%, 44.7%, 41.4%, 39.9%, 32.5%, and 37.6% of participants chose the highest level of health regarding each dimension, i.e. Physical functioning, Role limitations, Social functioning, Pain, Mental health and Vitality. Meanwhile, 24% of participants chose the worst level of health in the Role limiting dimension. Overall, 2.5% participants scored the full health (‘111,111’) and 0.1% scored the worst health (‘645,655’) based on the SF-6D health state classification system. Figure 1 presented the distribution of SF-6D overall score or the so-called utility value of the study population. The skewness coefficient and kurtosis coefficient of the utility were -0.255 and 0.775.

**Table 2:** Frequencies of each level of six dimensions of SF-6D

Dimension	PF	RL	SF	BP	MH	VT
Level 1	17.6	44.7	41.4	32.5	24.3	16.1
Level 2	47.3	26.8	22.0	4.3	39.9	37.6
Level 3	19.3	4.5	20.4	31.7	21.1	30.4
Level 4	4.6	24.0	10.7	16.7	11.7	12.0
Level 5	8.8	5.6	11.4	3.0	3.9	
Level 6	2.5	3.4				

*PF* physical function, *RL* role limitation, *SF* social function, *BP* body pain, *MH* mental health, *VT* vitality

### Differences in SF-6D overall score across subgroups

Table 3 showed the differences in SF-6D overall score across subgroups. The mean score of SF-6D utility value was 0.743 (SD: 0.141) and the median was 0.756 [Interquartile range 0.632–0.859]. The relatively low scores were presented in participants who were female, not educated, not married, living alone, and who had low household income, having high health expenditure, with more comorbidities and having no hypertension (p < 0.01).

**Table 3:** Differences in Health State Utility scores across subgroups (n− = 933)

Variables	Mean(95%Cl)	SD	Median	IQR
*All*				
*Gender *	0.743 (0.734–0.753)	0.141	0.756	0.632–0.859
Male	0.782 (0.768–0.795)	0.131	0.801	0.700–0.884
Female	0.718 (0.707–0.730)	0.141	0.725	0.600–0.828
Age(years)*				
50–59	0.765 (0.736–0.793)	0.133	0.778	0.706–0.863
60–69	0.757 (0.744–0.771)	0.138	0.769	0.648–0.873
70–79	0.728 (0.713–0.743)	0.142	0.736	0.611–0.843
> 80	0.705 (0.670–0.741)	0.156	0.728	0.563–0.834
Education*				
Illiteracy	0.715 (0.702–0.728)	0.142	0.719	0.596–0.832
Primary school	0.757 (0.741–0.773)	0.138	0.770	0.654–0.867
Junior school	0.788 (0.769–0.808)	0.125	0.807	0.716–0.882
Senior school or above	0.783 (0.739–0.827)	0.144	0.815	0.667–0.886
Marital status*				
Married	0.747 (0.737–0.757)	0.141	0.762	0.638–0.863
Others	0.724 (0.701–0.746)	0.138	0.730	0.610–0.838
Way of residence*				
Living alone	0.711 (0.685–0.736)	0.138	0.714	0.609–0.821
Living with a spouse	0.738 (0.726–0.750)	0.144	0.757	0.617–0.856
Others	0.774 (0.757–0.790)	0.129	0.786	0.685–0.874
*Occupation *				
Famers	0.734 (0.725–0.744)	0.138	0.745	0.623–0.849
Others	0.784 (0.761–0.806)	0.148	0.807	0.671–0.911
Household income (RMB Yuan)*				
< 5000	0.708 (0.693–0.723)	0.142	0.716	0.582–0.819
5000–9999	0.731 (0.711–0.751)	0.136	0.742	0.607–0.840
10,000–14,999	0.774 (0.753–0.796)	0.127	0.778	0.704–0.872
> 15,000	0.779 (0.763–0.796)	0.137	0.799	0.675–0.890
Health expenditure (RMB Yuan)*				
< 2000	0.776 (0.762–0.790)	0.137	0.801	0.674–0.877
2000–3999	0.737 (0.717–0.757)	0.139	0.749	0.628–0.854
4000–5999	0.726 (0.699–0.753)	0.148	0.722	0.604–0.844
> 6000	0.708 (0.692–0.725)	0.134	0.718	0.600–0.815
Comorbid diseases*				
1	0.776 (0.765–0.787)	0.131	0.793	0.689–0.874
2	0.705 (0.689–0.721)	0.141	0.716	0.594–0.813
> 3	0.664 (0.633–0.694)	0.139	0.649	0.553–0.791
*Health insurance *				
No	0.708 (0.646–0.771)	0.155	0.676	0.570–0.858
Yes	0.744 (0.735–0.754)	0.140	0.758	0.633–0.859
Hypertension stages*				
Normotensive	0.695 (0.678–0.712)	0.142	0.705	0.625–0.858
I	0.749 (0.736–0.763)	0.138	0.758	0.638–0.867
II	0.774 (0.755–0.794)	0.130	0.784	0.633–0.839
III	0.818 (0.790–0.846)	0.120	0.835	0.631–0.857
*Duration of hypertension *				
< 5	0.752 (0.736–0.768)	0.144	0.763	0.650–0.870
5–9	0.751 (0.735–0.768)	0.140	0.766	0.638–0.868
10–14	0.730 (0.708–0.752)	0.138	0.745	0.603–0.837
> 15	0.729 (0.709–0.748)	0.138	0.738	0.611–0.838
*Non-pharmaceutical therapies *				
*Exercise *				
No	0.742 (0.729–0.755)	0.143	0.758	0.648–0.859
Yes	0.745 (0.733–0.758)	0.139	0.753	0.642–0.856
*Alimentary control *				
No	0.746 (0.723–0.770)	0.147	0.767	0.641–0.867
Yes	0.743 (0.733–0.752)	0.140	0.755	0.630–0.857
*Work-rest schedule *				
No	0.731 (0.709–0.754)	0.142	0.749	0.606–0.839
Yes	0.746 (0.736–0.756)	0.141	0.757	0.638–0.863
*Emotion control *				
No	0.744 (0.720–0.768)	0.143	0.761	0.639–0.856
Yes	0.743 (0.734–0.753)	0.141	0.755	0.631–0.860
*Pharmaceutical therapies *				
*Take medicine in one year *				
No	0.747 (0.720–0.774)	0.147	0.745	0.648–0.867
Yes	0.743 (0.733–0.753)	0.140	0.757	0.630–0.857
*Take medicine in two weeks *				
No	0.747 (0.727–0.766)	0.144	0.754	0.646–0.858
Yes	0.743 (0.732–0.752)	0.140	0.757	0.630–0.859
*Pharmaceutical Compliance *				
*Forget taking medicine*				
No	0.741 (0.729–0.753)	0.141	0.749	0.627–0.856
Yes	0.748 (0.732–0.764)	0.142	0.764	0.648–0.862
*Drug discontinuation by patients themselves *				
No	0.744 (0.734–0.755)	0.140	0.757	0.637–0.857
Yes	0.739 (0.715–0.762)	0.147	0.758	0.598–0.860
*Drug discontinuation for improvement *				
No	0.740 (0.728–0.752)	0.141	0.747	0.617–0.857
Yes	0.750 (0.734–0.766)	0.142	0.767	0.650–0.859
*Drug discontinuation for deterioration *				
No	0.742 (0.731–0.752)	0.141	0.756	0.628–0.857
Yes	0.750 (0.727–0.773)	0.143	0.762	0.650–0.864

ANOVA* p < 0.01

### Associated factors of SF-6D utility score

Table 4 presented the results of multivariate linear regression analyses. Being female, having two or more comorbidities, and having higher than 2000 RMB health expenditure were associated with lower SF-6D overall score compared with their peer in the reference groups. Being a farmer, having stage I and above hypertension, having higher household income were associated with higher SF-6D overall score compared with the reference groups.

**Table 4:** Associated factors of SF-6D utilities by multivariate regression model (n = 933)

Variables	Coefficient	SE
*(Constant)*	0.811	0.046
*Gender (ref: Male)*		
Female	− 0.046**	0.010
*Age (ref:50–59)*		
60–69	0.016	0.015
70–79	0.000	0.016
> 80	− 0.025	0.025
*Education (ref: Illiteracy)*		
Primary	− 0.001	0.010
Junior	0.006*	0.013
Senior or above	− 0.017	0.021
*Marital status (ref: Married)*		
Others	− 0.019	0.019
*Occupation (ref: Others)*		
Famers	0.032**	0.011
*Way of residence (ref: Living alone)*		
Living with a spouse	0.016	0.013
Others	0.017	0.016
*Health insurance (ref: Yes)*		
No	− 0.030	0.024
*Comorbid diseases (ref:1)*		
2	− 0.066**	0.009
> 3	− 0.098**	0.015
*Hypertension stages (ref: Normotensive)*		
Stage I	0.047**	0.010
Stage II	0.079**	0.013
Stage III	0.095**	0.016
*Health expenditure (RMB) (ref: < 2000)*		
2000–3999	− 0.042**	0.011
4000–5999	− 0.046**	0.013
> 6000	− 0.071**	0.010
*Household income (RMB) (ref: < 5000)*		
5000–9999	0.015	0.012
10,000–14,999	0.045*	0.013
> 15,000	0.064*	0.011

*p < 0.05 ;**p < 0.01; *SE* Standard Err

## Discussion

Rural population in China generally have relatively poor material condition and relatively low educational level and health literacy [37, 38]. Chronic conditions and challenges due to aging are common among this population [34, 34]. However, the access to and the use of health care services and health insurance are limited [36]. In terms of hypertension management, rural population are less likely aware of hypertension early signs and less likely seek for medical help and adhere to medical treatment plan [37, 38]. Taken the above features into account, rural population may be at risk of suboptimal health-related quality of life. Therefore, it is crucial to extend the understand of associated factors of quality of life among patients with hypertension in rural area in China.

We have examined the utility of SF-6D among a community-dwelled participant in rural China. SF-6D overall score was significantly different across subgroups regarding gender, education, marriage, way of residence, household income, health expenditure, comorbidities, and hypertension stage. Gender, occupation, health expenditure, household income, comorbidities, and hypertension stage were associated with higher or lower SF-6D overall score.

We found that the utility value of SF-6D in the present study was lower than that of EQ-5D when these two instruments were used to measure HRQOL of patients with hypertension[20, 33]. The mean utility value of SF-6D in our study (i.e. 0.743) was lower than the mean utility value of EQ-5D among participants with hypertension reported by two previous studies (i.e. 0.921 and 0.850). It was also lower than the normative value of SF-6D measured in Hongkong population with hypertension (i.e. 0.746) [39]. Our study did not suggest the floor effect of SF-6D, which was not consistent with the study by Ferreira et al. [40]. The differences in the utility values across subgroups may be due to different instruments to measure quality of life. EQ-5D mainly focuses on measuring the physical domain of quality of life. There are four dimensions reflecting physical aspects and one dimension is about mental health. However, SF-6D is more comprehensive, reflecting the quality of life from physical, psychological, social and emotional aspects, which was a suitable instrument to measure the impacts of hypertension on patient's health. As a chronic disease, hypertension may not have some influences on physical health that patients can feel directly, but taking medication on a daily base and concerns about other complications due to hypertension may increase the psychological burden.

We found that higher educational level was associated with higher utility value that can be interpreted as better HRQOL, which was consistent with previous studies by Zhou et al. [41], and Andrade et al. [42]. The present study did not show that older age was associated with lower HRQOL score among the hypertensive individuals. A previous study indicated that comorbid diseases might influence disease severity and body pain/discomfort [43], which could result in worse health states. Two study results may be related to participants differences.

The present study found that household income was associated with poorer HRQoL. Zhang L et al. suggest that people with low household income tended to have higher healthcare needs but lacked the financial capacity to pay for health services [20]. High health expenditure is generally considered as an economic burden to the household, and it often places households at higher risk of catastrophic health expenditure and pushes them into poverty, which in turn inevitably has a negative impact on people’s HRQoL.

We did not find significant association between marital status and HRQOL, which was inconsistent with a previous study [19]. Zhang et al. showed that marital status was one of the factors to impact the HRQoL of patients with hypertension and married patients reported higher HRQoL than divorced or widowed patients. In our study, a large proportion of people were married, it was hard to observe the differences in HRQOL across marital subgroups.

As reported by previous studies, normally the individual with higher stage hypertension had lower HRQOL [44, 45]. However, the present study found the opposite result that the patients with higher stage hypertension reported higher HRQOL scores. This may be explained by two reasons. First, hypertension is a chronic disease with a long course. It may be asymptomatic or not obvious at the early stage, leading to patients' unawareness [46]. As the disease progresses, symptoms begin to appear and begin to affect the patient's feelings and quality of life. In rural China, the elderly usually has a low level of education, poor understanding of the prognosis of hypertension, and generally do not pay attention to the treatment of hypertension unless symptoms occur. Only patients with severe symptoms of hypertension, which maybe has already affected their daily life functioning and quality of life, may seek help in terms of hypertension treatment. This may partly explain why patients with better blood pressure control in this study had a relatively low quality of life. In addition, some patients maybe did not perceive hypertension as a health condition that can cause severe complications without proper treatment, so they did not adhere to the treatment and maybe rated their quality of life to the normal level or better, which may explain the association between high blood pressure with better quality of life. This finding indicated that it was necessary to educate the patients regarding the knowledge of prognosis of hypertension and arouse the attention of patients to importance of early control of blood pressure, in order to achieve the effective control of BP. Second, the severity of hypertension might affect people’s perception of their general health, which might influence how they rated their HRQoL. They may rate their HRQOL better than normotensive individuals after successfully controlling the blood pressure or by developing good coping skills. We recommended studies in the future to confirm or deny this finding in our study.

## Strengths and limitations

The present study has randomly selected a large community-based sample of middle and old aged patients with hypertension from the community population in rural areas in Shandong province, China. Additionally, we have measured a comprehensive set of potential associated factors of HRQOL.

Our study has some limitations that deserved to mention. First, as a cross-sectional design, the association between HRQoL and factors cannot be interpreted as causal. Second, there might be other potential factors that affect HRQoL, such as medication adherence, hospital admission, which might cause a deviation of the results. However, we did not measure in our study. Third, EQ-5D has not been investigated in this paper, so we were not able to compare the utility value measured by SF-6D with the widely-used EQ-5D. Fourth, the age groups in this study were middle and old age in a rural area. This may influence the generalization of our results to the general population. In the selected counties in our study, residents who were under 50 years normally were not available for the survey because they mostly immigrated to the cities and worked there. Fifth, given the social, cultural, geographic and economic characteristics of Shandong Province, the selected sample in our study could not fully represent the rural population in China, which may limit the generalization of the findings from the present study. We recommended further similar studies to be conducted among larger and more representative samples in China by selecting random samples from North, South, East and West China.

In conclusion, the present study was the first to apply SF-6D to measure quality of life among a large, representative community-based sample of patients with hypertension in rural area in China. SF-6D was capable to measure quality of life of Chinese rural population. And multiple factors were associated with participant’s quality of life. Being female, having two or more comorbidities and the health expenditure higher than 2000 RMB were associated with worse quality of life; while being farmer, having high household income and having stage I and above hypertension were associated with better quality of life.

## Data Availability

The data supporting the conclusion of this article are includes within the article. Any queries regarding these data may be directed to the corresponding author.
